# Enabling Demonstrated Consent for Biobanking with Blockchain and Generative AI

**DOI:** 10.1080/15265161.2024.2416117

**Published:** 2024-11-05

**Authors:** Caspar Barnes, Mateo Riobo Aboy, Timo Minssen, Jemima Winifred Allen, Brian D. Earp, Julian Savulescu, Sebastian Porsdam Mann

**Affiliations:** a Harvard Medical School; b AminoChain, Inc; c University of Cambridge; d Stanford University; e University of Copenhagen; f Monash University; g University of Oxford; h National University of Singapore

**Keywords:** Informed consent, biomedical research, research ethics, blockchain, generative AI, large language models (LLMs)

## Abstract

Participation in research is supposed to be voluntary and informed. Yet it is difficult to ensure people are adequately informed about the potential uses of their biological materials when they donate samples for future research. We propose a novel consent framework which we call “demonstrated consent” that leverages blockchain technology and generative AI to address this problem. In a demonstrated consent model, each donated sample is associated with a unique non-fungible token (NFT) on a blockchain, which records in its metadata information about the planned and past uses of the sample in research, and is updated with each use of the sample. This information is accessible to a large language model (LLM) customized to present this information in an understandable and interactive manner. Thus, our model uses blockchain and generative AI technologies to track, make available, and explain information regarding planned and past uses of donated samples.

## INTRODUCTION

Effective medical practice and the advancement of biomedical knowledge depend on high quality research. Such research often involves risks which participants may not be able to foresee. The stakes and complexity of biomedical research, as well as informational, power, or other asymmetries between researcher and subject, has at times led to exploitation and unjustifiable harm (Beecher 1966; Gere 2020; Fish et al. 2021). Consequently, professional norms (Chisholm and Askham 2006), ethical and regulatory codes (World Medical Association 2013; Arellano et al. 2018) as well as domestic (Rumbold and Pierscionek 2017; Arellano et al. 2018) and international law (UN General Assembly 1966) all recognize duties not to subject anyone to medical experimentation unless, among other criteria, they have given valid consent: i.e., permission or agreement that is voluntary (e.g., free from coercion) and based on an adequate understanding of the risks and benefits involved (Nelson et al. 2011).

Originally introduced to facilitate treatment compliance while protecting patients from undue harm, the current ethical and legal justification for informed consent requirements primarily focus on respect for individual autonomy (Sheehan 2011; O’Neill 2002; Beauchamp and Childress 2019) and shielding individuals and institutions from liability under tort law (Koch 2018).

In interventional research with significant potential risks to health, adequately informed, study-specific consent is essential (Maloy and Bass 2020). However, some types of research may not fit this ideal (Porsdam Mann, Savulescu, and Sahakian 2016). Biobanking, for instance, is a practice whereby biological materials such as blood or tissue samples as well as associated data are stored and preserved for use in future research. Unlike clinical trials, biobanking can lack predefined protocols and participant groups. At donation, the future uses of samples are often unknown to all parties, making traditional justifications and operationalizations of informed consent limiting and inappropriate (Mikkelsen et al. 2019). Contacting donors for consent for each use is also infeasible due to the vast number of samples stored (Kaye et al. 2012; Steinsbekk, Kåre Myskja, and Solberg 2013).

Several modifications of study-specific consent have been proposed to address these issues in the biobanking context. Initial suggestions included one-off “open” or “blanket” consent for any future research, and “broad” consent for a range of studies with certain restrictions. However, both of these proposals have faced significant criticism for entailing limited ethical oversight and regulatory control (Hallinan and Friedewald 2015; Steinsbekk, Kåre Myskja, and Solberg 2013; Wendler 2006, 2013). As a result, a new generation of consent procedures has been proposed. These include “tiered” consent, which separates research uses into categories of varying risk and sensitivity (Steinsbekk, Kåre Myskja, and Solberg 2013); “meta-consent,” in which participants are asked to state their preferences regarding being asked for consent for future research (and thus consenting to future consent requests, rather than to the research itself) (Ploug and Holm 2016); and “dynamic” consent platforms that use Web 2.0 interactive webpages to allow donors to tailor and update their consent preferences in real time as their samples are requested for new studies (Kaye et al. 2015; Budin-Ljøsne et al. 2017).

In this paper, we introduce and describe the novel concept of *demonstrated consent*. Demonstrated consent is a biobanking consent- and inventory-management system based on the technology known as blockchain (described below), bolstered by advances in generative artificial intelligence (AI). The proposed system allows individual donors to see the ways in which their samples have been and are being put to use by associating unique non-fungible tokens (NFTs; i.e. Kostick-Quenet et al. 2022) to donated samples. Essentially, this means making up-to-date and study-specific information of the kind relevant to consent (e.g., hypotheses, protocols, risks) available to sample donors such that they may, to the extent that they desire, inform themselves about their sample’s actual usage and revoke or modify consent if they wish.

Blockchain is an emerging technology that consists of cryptographically linked blocks of data distributed across a network of nodes that validate and record transactions, which allows for secure, transparent, and auditable exchanges of information and values without the need for intermediaries. Blockchain technology has various affordances useful for biobanking, including the ability to execute smart contracts. Smart contracts are programs running “on top” of blockchains that self-execute when certain conditions are met. Another affordance is the ability to integrate recent advances in generative AI, in particular large language models (LLMs), which have recently become familiar to a broader audience following the release of ChatGPT by OpenAI. In the context of the current proposal, LLMs could be used to convey information about the use of donated samples to donors in a personalized, interactive manner.

We propose a specific technical implementation of a demonstrated consent system leveraging generative AI and blockchain technologies. This system, we argue, represents a distinct model that combines the benefits of several existing consent frameworks while mitigating some of their limitations. As in broad consent, demonstrated consent involves an initial consent procedure in which the use of samples and associated data for specific fields of research is authorized. Like dynamic and meta-consent, demonstrated consent involves a user-friendly online platform which allows individuals to update their consent preferences should they so desire. Unlike these models, however, demonstrated consent does not require donors to actively manage their preferences on an ongoing basis. Instead, it provides a secure, transparent, up-to-date, and easily accessible repository of information that donors can engage with at their own pace and to the extent that they wish. In this way, demonstrated consent empowers donors to make informed decisions about their participation in biobanking research without the risk of choice overload or consent fatigue. In demonstrated consent, as in traditional consent models, a participant would still have their initial willingness to participate in biobanking research assessed by a human researcher. Unlike traditional consent models, under demonstrated consent the participant would be able to access at will a source of information about the actual current and planned future uses to which their materials are being, or will be, put.

We begin by briefly surveying the history of consent requirements and describing recent consent model proposals, focusing in particular on blanket, broad, tiered, meta, and dynamic consent. Next, we introduce the concept of demonstrated consent. We then describe an implementation of demonstrated consent using blockchain and generative AI technologies. To conclude, we encourage further discussion and use of demonstrated consent by relating its affordances and applications to the goals of informed consent, particularly in the broader context of facilitating ethical research practices.

## HISTORICAL AND CURRENT CONSENT MODELS

The concept of informed consent has a long and complex history, with its roots tracing back to ancient Greek, Roman, and medieval thought (Lee 2018). However, the modern understanding and emphasis on informed consent in medical contexts is a relatively recent phenomenon. Throughout history, the justifications for obtaining consent have evolved, reflecting changes in social norms, scientific advancements, and ethical considerations (Faden, Beauchamp, and King 1986). In medieval times, consent was primarily justified on the basis of beneficence, focusing on the benefits of patient cooperation for both patients and physicians (Faden, Beauchamp, and King 1986; O’Shea 2018). During the 17th to 19th centuries, legal considerations and professional duties began to shape consent requirements (Faden, Beauchamp, and King 1986; O’Shea 2018). The 20th century marked a significant shift toward the principle of respect for patient autonomy as the primary justification for informed consent (Gere 2020). This change was influenced by various factors, including Albert Neisser’s non-consensual medical experiments in Prussia (Vollmann and Winau 1996; Benedek 2014), the Nuremberg trials, increased legal interest in consent and liability, medical technological advancements, reports of unethical research practices including the Tuskegee and Guatemala syphilis studies (White and Reverby 2002), and social and civil rights movements. The Nuremberg Code, the Declaration of Helsinki, and the Belmont Report played crucial roles in establishing the central importance of respect for persons, autonomy, and informed consent in medical and research ethics, with beneficence and justice as secondary considerations (Gere 2020).

The Belmont Report and its so-called “mid-level” ethical principles—as they came to be known—were originally motivated by the need to protect participants in interventional and clinical research (Faden, Beauchamp, and King 1986). In the period between publication of the Belmont Report and the turn of the millennium, tissue samples were often regarded merely as objects associated with particular diseases or medical procedures, not as extensions of the individual donors themselves (Hoeyer 2008). In the late 1990s, however, the confluence of patient advocacy, large-scale genomic projects, and the increasing commercialization of research resulted in a reconceptualization of the storage and use of samples as normative issues with important ethical implications (Hoeyer 2008). By the mid-2000s, the use and storage of tissue samples for research purposes was generally understood to require informed consent (Wendler 2006).

### Broad Consent for Biobanking

Commentators noted that the informed consent paradigm used for interventional and clinical research may be infeasible in the more open-ended context of biobanking (Wendler 2006). This realization led to calls for less onerous adaptations of informed consent for biobanking, notably “blanket” and “broad” consent. Where the former involved a one-off consent for unrestricted future uses of a sample, the latter likewise allowed for a single consent for future uses of samples, albeit with some restrictions relating *inter alia* to governance, risk, and Institutional Review Board (IRB) oversight (Boers, van Delden, and Bredenoord 2015; Wendler 2006). These proposals aimed to balance an increased emphasis on individual autonomy with beneficence-related concerns about the ability of researchers to establish safety and efficacy for new potential treatments.

Yet, the very feature motivating the adoption of broad consent—reducing the onerous requirements for re-consenting—sparked concerns about whether broad consent could genuinely respect the autonomy of participants (Minssen and Schovsbo 2014). By not providing detailed information about each specific future use of biobanked samples, broad consent might not enable participants to make adequately informed decisions aligned with their values and preferences. Several research groups noted that broad consent would best be able to balance the normative considerations at play if it involved not only governance and risk mitigation strategies but also the continuous provision of information about the ongoing use of samples (Grady et al. 2015; Mikkelsen et al. 2019; Wendler 2006).

### Alternative Biobanking Consent Models

Unfortunately, the continual provision of information appears to face similar feasibility and cost concerns as study-specific consent. In response to these challenges, a new generation of alternative consent models has been proposed (Minssen and Schovsbo 2014). Each of these alternatives seeks to increase the degree of involvement of donors in decisions concerning the use of their samples to a level above that of broad consent but below that of study-specific consent, yet they differ significantly in both the level of engagement sought and the means used to achieve it. While there are numerous alternative consent models, the discussion below will focus on three of the most widely discussed and implemented models, namely tiered consent, meta-consent, and dynamic consent (Loosman and Nickel 2022).

Tiered consent involves stratification of potential studies based on parameters such as risk or disease category (Wolf and Lo 2004). Tiered consent maintains the open-ended nature of broad consent but restricts the operation of this consent to the specific areas chosen at the point of consent. For example, a donor might specify that their consent is valid for all future cancer research but not to other types of research. This increases the degree and specificity of control by donors over the uses of their samples while still enabling future research in the areas consented to.

Meta-consent (Ploug and Holm 2016), like tiered consent, enables individuals to consent to specific areas of research. However, it goes beyond tiered consent by asking participants not what areas of future research they consent to, but rather when and how they would like to be asked for consent for future research. It essentially offers participants control over the consent process itself, including the option to decide on a case-by-case basis or to agree to broader categories of research, thereby addressing the issue of autonomy more directly by empowering individuals to shape their own consent experience.

Dynamic consent (Kaye et al. 2015; Budin-Ljøsne et al. 2017) incorporates elements of all the proposals discussed so far. It involves the use of web-based platforms that allow donors to specify and then continually update their consent preferences. This means that it can be seen as a technical implementation of various other consent types. For example, a dynamic consent portal could be used to request study-specific consent or to allow individuals to express their meta- or tiered-consent preferences. Most importantly, this proposal is “dynamic” because it allows individuals to update their consent preferences should these change (Kaye and Prictor 2019).

### Criteria for Biobanking Consent Models

While all three models significantly enhance participant autonomy by providing a more flexible and participant-centered approach than broad consent, they are also all vulnerable to the same types of objections that were originally levied against study-specific consent. Mikkelsen et al. (2019, 5–6) provide a detailed critique of these models by referencing three criteria which, they argue, a consent model for biobanking should ideally fulfill:

- “Information Criterion: Inform potential participants about the relevant risks and benefits of biobank research (especially informational risk) so that [they] can make an autonomous choice about participation in light of this information;
- Value Criterion: Offer participants the opportunity to assess whether the research to be conducted with biobank materials is in line with their personal values, and to make consent decisions based on this; [and]
- Duration Criterion: Afford ethical protection for the duration of the subject’s participation in the biobank, and give the subject a real and meaningful opportunity to reassess [their] consent for that duration and an actionable right of withdrawal (provided, of course, that the participant is still alive and competent).”

While we largely agree with Mikkelsen et al.’s criteria, we argue that consent systems for biobanking should not seek only to maximize individual donor autonomy but should aim for an appropriate *balance* between respect for autonomy and other core principles of bioethics: for example, minimizing undue risk of harm to individual patients or research participants (non-maleficence); beneficence, including in relation to the long-term fruits of medical and scientific research (i.e., enabling medicine to fulfill its beneficence-based obligations to the wider community, such as by discovering or improving upon treatments); and justice, including—but not limited to—the fair and equitable distribution of these very fruits.

Of course, by invoking these traditional bioethical principles in the context of biobanking, we do not mean to suggest they are exhaustive of the moral considerations that must be taken into account when evaluating the ethics of research in this area (Baker 2024; Beauchamp & Childress, 2019; Herranz 1997; Huxtable 2013; Takala 2001). For example, some have proposed that *solidarity* should be given greater prominence in the field (Prainsack and Buyx 2012). In fact, as Robert Baker (2024) argues, even the current, narrow notion of “respect for autonomy” was (and remains) a controversial substitution for a broader notion of “respect for persons” that had been articulated in earlier drafts of the Belmont Report. And it was this earlier and more expansive notion that he suggests was at the heart of the founding of bioethics as a field, i.e., as a way to address socio-structural rather than individual-level concerns, including the systemic racism brought to light by the Tuskegee syphilis experiments. As he writes, respect for persons is “an inherently communal concept that extends beyond the first-person singular self to include other persons and is congruent with ideals of family, the common good, solidarity, and community” (4). In line with this analysis, we argue that consent frameworks must, at a minimum, be evaluated not *only* in terms of their impacts on the individual donor but also in terms of their impacts on the common good. For this reason, also mindful of other bioethical principles apart from autonomy (e.g., justice), we propose a fourth criterion:

Balance Criterion: Balance the protection of individual autonomy with wider societal interests in the progress of science and medicine, while also honoring the demands of justice.

The balance criterion is implicit in the argumentation of Mikkelsen et al., yet its significance, we believe, is such that it warrants independent consideration as a substantive criterion for evaluating the ethical implications of consent models in biobanking.

### Practical Challenges of Current Consent Models

Mikkelsen et al. (2019) underscore the inherent difficulties that study-specific consent and the three alternative consent models—tiered, meta-consent, and dynamic consent—face in fully satisfying their original ethical criteria. Study-specific consent, while theoretically offering the highest level of specificity and respect for autonomy, struggles with feasibility and cost in the biobanking context. Its focus on the risks and benefits of individual studies also fails to account for the broader informational risks inherent in biobanking governance and policy.

Dynamic consent, despite its innovative approach to addressing these issues through technology, cannot escape the fundamental challenges of study-specific consent when used in this capacity. Mikkelsen et al. (2019) point out that a primary benefit of dynamic consent is its ability to implement study-specific consent or other types of consent via a technological platform. To the extent that this is the case, the model is vulnerable to the same objections levied against those consent models: While the use of a technological platform undoubtedly increases the efficiency of study-specific consent, the costs involved can still be prohibitive due to their likely impact on scientific and medical progress and thus on beneficence as embedded in our newly proposed balance criterion. Like study-specific consent, dynamic consent may also fail to focus on the type of informational risks characterizing biobanking generally (as opposed to any one study in particular).

Tiered and meta-consent models attempt to refine the consent process by allowing participants to choose from predefined categories of research or consent modalities. However, these models face challenges in accurately defining and maintaining clear boundaries between categories as research evolves. The difficulty in anticipating all future research directions means that the specificity promised by these models may diminish over time, potentially misaligning with participants’ values and expectations. Furthermore, the categorization process itself may not fully capture or respect the diversity of participant values, particularly for historically disenfranchized groups, raising concerns about fairness and inclusiveness (Mikkelsen et al. 2019).

To these arguments may be added other important considerations. Advances in cognitive psychology and behavioral economics have called into question the implicit assumption that more choice is always better. Paradoxically, too much information and choice can lead to decision paralysis, demotivation, and reduced satisfaction with choices made (see Ram 2008; for a review and meta-analysis of empirical findings, see Chernev, Böckenholt, and Goodman 2015; for important qualifications on generalizability derived from cross-cultural research, see Reutskaja et al. 2022). This phenomenon, often referred to as “choice overload,” suggests that an increase in options, while theoretically enhancing freedom and autonomy, can in practice complicate decision-making processes and lead to worse outcomes for individuals. A related phenomenon is known as consent fatigue: Stronger requirements or protections may counter-intuitively lead to weaker consent due to the tendency for repeated requests to reduce the motivation and depth of processing related to each individual token of consent (for example, to manage cookie preferences on websites) (Schermer, Custers, and Van der Hof 2014).

If such insights continue to prove robust and reliable in relevant populations and decision-making domains, they have profound implications for the consent models discussed in the context of biobanking. The belief that providing participants with a wide array of consent options (such as those offered by tiered, meta, and dynamic consent models) would empower them and enhance their autonomy is nuanced by these findings. While these models aim to cater to diverse participant preferences and values, they also risk overwhelming participants with complex decisions. This complexity may not only hinder participants’ ability to make informed choices but can also lead to dissatisfaction or regret, undermining the very autonomy these models seek to promote.

These considerations lead Mikkelsen et al. (2019) to advocate a return to broad consent as the best option for biobank consenting—provided, as they say, that such consent is not only broad but also deep. This depth, they argue, entails a continuous engagement with participants, providing them with ongoing updates about the use of their samples and the outcomes of related research. Such an approach would theoretically combine the wide scope of broad consent, minimizing operational burdens on biobanking activities, with a level of participant engagement and information provision more akin to what is envisioned in dynamic consent models. However, it is crucial that this “deep” broad consent does not inadvertently contribute to the choice overload and consent fatigue concerns identified earlier. Instead, the provision of information must be carefully managed to enhance understanding and maintain participant satisfaction without overwhelming them. Specifically, Mikkelsen et al. advocate a once-yearly provision of summary information about ongoing research uses of donated samples.

While we agree with the reasoning of Mikkelsen et al., we propose what we see as an improved method of ensuring the relevant depth of consent: demonstrated consent based on blockchain technologies appropriately integrated with generative AI. In addition to satisfying the depth requirement for broad consent to be adequately informed and protective of donor interests and values, demonstrated consent has several additional advantages related to its technical implementation. These include, but are not limited to, enabling “prosenting” (roughly, a mechanism for donors to preauthorize data access based on pre-specified criteria; see Porsdam Mann et al. 2021) and the automation of several aspects crucial to efficient biobanking, such as inventory management and material transfer agreements.

## DEMONSTRATED CONSENT

Demonstrated consent is a novel consent framework that combines the scope of traditional broad consent with the ongoing participant engagement and information provision envisioned in “deep” broad consent models. It leverages blockchain technology and non-fungible tokens (NFTs) to create a transparent, auditable, and participant-centric system for managing consent and sample usage in biobanking. Demonstrated consent involves the association of each donated sample with a unique NFT, which serves as a digital representation of the sample on a blockchain. In the context of biobanking, an NFT would represent a specific donated sample, with the token’s metadata containing information about the sample’s characteristics, storage location, and associated consent preferences. This metadata, as well as study protocols and other study-specific information, is accessible by an integrated LLM adapted (via fine-tuning or the use of custom instructions) to the specific task of explaining that information in a way that is understandable by and desirable to individual donors.

Demonstrated consent involves three core features:

1. An initial *personalized* consent for future research use of donated samples—broad as default but can be modified.
2. Access to an automatically updated, privacy-preserving, and reliable source of near-real-time information about the past, present, and potential future uses of donated materials.
3. A mechanism for altering broad consent preferences, for reverting to any other type of consent model, and for withdrawing consent.

These features map directly onto the criteria for broad consent to be ethically desirable identified in previous research (Grady et al. 2015; Mikkelsen et al. 2019; Wendler 2006). They also enable those individuals who wish to specify more details about their consent preferences—akin to meta- and dynamic consent—to do so. The key distinguishing feature of demonstrated consent is the ongoing accessibility or provision of relevant information in accordance with the donor’s preferences. This is achieved partially through a blockchain-based tracking mechanism and partially through LLM integration (see section IV below).

When a participant donates a sample to a biobank, they would specify their broad consent preferences, including any restrictions on the types of research for which their sample can be used. This consent information would be encoded into the metadata of an NFT associated with each sample, creating an immutable record of the participant’s wishes. As researchers request access to samples for specific studies, smart contracts would automatically verify that the requested usage aligns with the consent preferences associated with each sample’s NFT.

The key innovation of demonstrated consent lies in its approach to ongoing participant engagement and information provision. As samples are used in research studies, the associated NFTs would be updated with metadata about each (subsequent) usage, creating a transparent record of how the samples have been used over time. Participants would have access to a user-friendly interface that allows them to view the complete history of their sample’s usage, including the types of studies conducted, the research institutions involved, and any resulting publications or findings, and to interact with a dedicated LLM to have this information explained to them (see section IV.II below). Importantly, while this interface would always be available to donors, there would at no time be a requirement for them to interact with the system if they do not desire to do so.

This ongoing information availability serves several purposes. First, it enhances participant autonomy by providing individuals with a clearly understandable record of how their samples are being used, allowing them to make informed decisions about their continued participation. Second, it fosters trust between participants and researchers by demonstrating the biobank’s commitment to transparency and accountability. Finally, it may encourage greater participation in biobanking by giving individuals a tangible sense of their contribution to scientific progress.

In addition to its consent management features, demonstrated consent also offers practical benefits for biobanks and researchers. By storing sample metadata on a blockchain, biobanks can create a secure, decentralized inventory management system that facilitates sample tracking and reduces the risk of data loss or inconsistencies. Researchers can use the blockchain to quickly identify samples that meet their study criteria and verify that they have the necessary consent to access them, streamlining the sample acquisition process. The use of LLMs in this process also offers practical benefits for researchers, such as increased time-efficiency and auditable consent transcripts (Savage et al. 2024).

Demonstrated consent also enables the creation of a shared, interoperable data infrastructure for biobanking. By using a common blockchain platform and standardized NFT metadata schemas, biobanks can facilitate the secure and efficient sharing of samples and data across institutions, fostering collaboration and accelerating research progress. This increased interoperability aligns with the principle of beneficence by maximizing the utility of donated samples and promoting the advancement of scientific knowledge.

Furthermore, the transparency and auditability provided by the blockchain can help to identify and address any disparities in sample usage or research focus, helping to ensure that underrepresented populations are not left behind, in accordance with the demands of justice. Additionally, the decentralized nature of the blockchain enables the creation of community-driven biobanks, empowering marginalized communities to have a greater say in how their samples are used and how the benefits of research are shared.

## IMPLEMENTING DEMONSTRATED CONSENT WITH BLOCKCHAIN AND GENERATIVE AI

The implementation of demonstrated consent relies on the integration of two key technologies: blockchain and generative AI, specifically LLMs. These technologies would work in tandem to create a secure, transparent, and user-friendly system that makes available to donors the information necessary to make informed decisions about their ongoing participation in biobanking research. In this section, we describe how blockchain and LLMs can be leveraged to enable the core features of demonstrated consent.

### Blockchain

‘Blockchain’ refers to a set of advances in computer science and cryptography which collectively enable data and value transactions without the need for trusted intermediaries. A blockchain can be thought of as a shared digital ledger whose contents are synchronized across actors (‘nodes’) in a network. This ledger consists of a series of data “blocks” containing all transactions within a given time period (typically, minutes) which are sequentially connected (‘chained’) to each other. Each block contains a timestamp and a hash (the outcome of a cryptographic function that produces a fixed-size string of characters unique to the data it represents, serving as a digital fingerprint of that data) of the previous block, features designed to ensure the integrity of the record. Before a block is added to the chain, nodes in the network must reach consensus as to the transactions or other information recorded within each block. This is achieved via a variety of game theoretic economic incentive structures, known as “consensus mechanisms,” which render tampering economically infeasible.

Originally introduced as the infrastructure underlying the cryptocurrency Bitcoin (Nakamoto 2008), blockchain technology has since been applied to a variety of non-financial use cases, including supply chain management, digital identity verification, and voting systems (Shrimali and Patel 2022). Notably, blockchain technology has several affordances of interest to healthcare and health research (Porsdam Mann et al. 2021; Vazirani et al. 2024). Among these are:

- Transparency, immutability and auditability: Once a transaction has been recorded on a blockchain, it is very difficult or impossible to change. The content of each block is visible to all nodes in a network. Blocks are added in near-real time.
- Decentralization: The state of the blockchain is shared with, and accessible by, all nodes in the network. This means that applications built on blockchains do not require trusted intermediaries (e.g. a bank, auditor, broker) to verify information in a transaction.
- Smart contracts: It is possible to create software which interacts with the information contained in a blockchain. Such programs are known as “smart contracts” because they have the ability to automatically execute agreements based on the information in a blockchain. For example, a smart contract could check a blockchain for the presence of consent and, if this is found, automatically release data in accordance with the consent parameters. Smart contracts are highly composable, and they can be integrated into non-blockchain technology stacks via application programming interfaces (APIs).

For these reasons, blockchain technology has been used in or proposed for a great variety of healthcare applications (Ghosh et al. 2023; Vazirani et al. 2019). Mamo et al. (2020), proposed a blockchain-powered framework for dynamic consent called Dwarna. Dwarna demonstrates the benefits of smart contracts, as their model incorporates mechanisms for patients to opt in to particular studies, withdraw consent, and erase existing data; a technicality that makes their model compliant with the European Union’s General Data Protection Regulation (GDPR) (Mamo et al. 2020).

Similarly, platforms like Genobank, Genomes.io, and LunaGenomics, demonstrate the utility of blockchain-based transparency, immutability, and auditability, as users can dynamically consent to sample and data use in response to direct requests from researchers. These platforms demonstrate blockchain technology’s decentralism; Genobank and Genomes.io, for instance, provides users with a “genomic vault” in which they can secure their genetic data on a blockchain, and where they are the sole owners and custodians of the data. LunaGenomics is a decentralized data aggregation platform and uses crypto-economic incentive mechanisms to pool user data for research use. From here, users can dynamically update their consent preferences for adding and removing their data to the pool.

These existing models share many features with demonstrated consent. However, unlike demonstrated consent, they are often limited to genomic data (in which individuals may have a personal interest), typically involve opting in, and do not provide any information about the actual usage of, or data derived from, one’s samples. By contrast, demonstrated consent is a novel framework that can be customized to suit the workflow of any type of bio-sample banking, at any level of decentralization. As we describe it here, demonstrated consent involves an initial customized consent (broad by default) followed by the ability to modify consent preferences. Demonstrated consent is powered by an inter-biobank network, connected by a blockchain platform operating on the back end (and thus invisible to both scientists and donors). A universal blockchain protocol could connect with a variety of biosample data sources (for instance, Lab Inventory Management Systems - LIMS, data capture softwares, pathology reports, bio-monitoring systems, etc.) to hold an inter-institutional aggregation of sample, patient, and research data provenance.

When a participant donates a sample to a biobank, information on the physical specimen may be stored in a LIMS system, and a blockchain connection here could create a unique NFT to represent the donated physical sample. This NFT’s metadata encodes information about the sample, including its characteristics, physical storage location, and donor consent preferences using a standardized schema, which is available for the donors to see. This means that donors may access information about their sample and its actual usage within studies (as opposed to only general information about the study). While our focus is on information management and consent, this standardization could enable smart contracts to automatically execute permissions for sample use or transfers between biobanks and researchers based on consent parameters.

The specific implementation of physical sample requests and distribution would depend on individual biobank practices. However, one way of handling this would be as follows. As researchers from different institutions request access to samples, smart contracts automatically verify that the requested usage aligns with the donors’ consent preferences, as encoded in the associated NFT metadata. If the conditions are met, access is granted, and the sample can be securely shared between biobanks using the blockchain infrastructure. Integrations with shipping information and bio-monitoring systems may hold the sample NFT in escrow until the physical sample arrives at the corresponding biobank. The use of smart contracts and standardized NFT metadata schemas streamlines the process of data sharing and collaboration, while ensuring that donors’ wishes are respected and regulatory requirements are met.

The blockchain keeps track of all requested and granted transfers as well as downstream uses of donated samples. As samples are transferred or used in research studies, the associated NFTs are updated with metadata about each usage via the lab’s LIMS. This creates a transparent record of the sample’s history and usage across biobanks and research institutions. Sample donors may access this record through a user-friendly interface that allows them to track the uses to which their samples are or will be put. This interface also enables donors to update their consent parameters or to withdraw from individual, planned studies.

While our proposal is agnostic to specific blockchain architectures, a pragmatic real-world implementation would likely involve a private, permissioned blockchain operated by a service provider. This provider would manage the blockchain backend, integrating it with existing biobank technology stacks. Researchers and biobanks would simply enter standard sample information into familiar software interfaces, with the service provider handling the technical aspects of NFT minting and blockchain operations. This approach minimizes additional requirements on existing stakeholders while providing the benefits of blockchain-based consent management.

### LLMs

Recent advances in generative AI, notably in large language models (LLMs), provide the other key enabling technology—in addition to blockchain—to address some of the perennial challenges of informed consent. Often, the complexities of biobanking consent involve intricate details concerning genetic information privacy, data protection, research programs and implications, and ethical considerations that may not be readily accessible to individuals without domain-specific regulatory and scientific training. In its original form, this information is often dense and highly technical in nature, and thus might not be understood by most people outside the relevant regulatory group or the immediate research team.

To address this informational challenge, we propose the design and deployment of an LLM system specifically fine-tuned for enhancing donor comprehension in demonstrated consent biobanking applications. The ability of LLMs to interactively generate and edit text in near real-time means that they are well-suited to explaining complicated topics (Porsdam Mann, Earp, Møller, et al. 2023). Their performance on specific tasks can be increased via a process called fine-tuning, in which the already-trained LLM is exposed to further, more targeted training on a more specific dataset. Such fine-tuned models can, but do not always (Zhang et al. 2024), outperform the underlying base model on particular tasks (Singhal et al. 2023).

In addition to fine-tuning for the provision of biobanking consent information, our proposed LLM system design leverages retrieval-augmented generation (RAG)—as described below—to help “translate,” explain, and assess comprehension of this multifaceted information so that it is customized and tailored to meet the unique information-processing needs and preferences of each potential donor. In essence, this design aims to harness the power of generative AI to bridge the information gap between biobanks, researchers, and donors.

Some of us (Allen et al. 2024) have recently proposed the use of LLMs to enhance the process of consenting patients in a clinical context, whereby the consent-seeking process would be supplemented by a fine-tuned LLM—in the form of a chat interface—that has access to up-to-date, procedure-specific information. As argued in Allen et al. (2024), the potential benefits of such an LLM-enhanced consent process include: reducing the explanatory burden on human consent-seekers (e.g., junior doctors); creating an automatic transcript of the consent conversation for human evaluation and quality control; heightened engagement with key information through the interactive conversational format; ability to test patient understanding of information; and ability to maintain up-to-date information through appropriate fine-tuning or information retrieval processes. Already, working prototypes of consent-facilitating LLMs are being tested in medical settings, with promising results (Aydin et al. 2023). A proof of concept using LLMs in consent for genomic research suggests that LLMs offer a satisfactory and time-efficient approach, without compromising participants’ understanding (Savage et al. 2024). Furthermore, this approach allows for the dynamic updating of information to reflect the latest developments and ethical standards in the field. Here, we advocate for an adaptation of this proposal for the biobanking context.

For purposes of blockchain-enabled demonstrated consent, our proposed LLM is fine-tuned and enhanced with additional information about a given biobank and its consent procedures. The fine-tuning focuses on the availability of information to donors in the biobanking context. Additionally, the LLM is enriched with a comprehensive database of information about the biobank, including biobank operations, consent protocols, FAQs, regulatory information, and ongoing research studies. This information is accessed through a vectorized knowledge database using the above-mentioned RAG approach (Lewis et al. 2020). This approach combines the power of pre-trained LLM (the generators) with a separate component (the retriever) that searches a database containing the collection of application and domain specific documents to find the relevant information based on the user input query (i.e., the donor question in this context). Thus, by using RAG, the LLM produces an output that not only reflects the understanding encapsulated within its model weights (based on general pre-training and fine-tuning for biobanking) but also incorporates internal specific information for a particular biobank. This makes the output more relevant to the donor’s specific queries, accurate, and informative.

While general LLMs are known to face challenges such as hallucinations, bias, and confidentiality concerns, these issues are significantly mitigated in our proposed system. The use of RAG provides the LLM with access to “ground truth” in the form of study-specific documents, substantially reducing the risk of hallucinations. Bias, while still a concern, is less pronounced when the model is working with factual, domain-specific information. Nevertheless, thorough testing of the LLM component before implementation would be crucial to further minimize these risks. In general LLMs, confidentiality and privacy can be key issues; however, the type of study-specific information we envision the system handling, such as research protocols, should already be publicly available, thus alleviating this concern.

The RAG component provides flexibility and scalability: in many cases, the trained biobank LLM can be adapted to different biobanks by updating the knowledge sources (i.e., the specific biobank consent database) without the need for retraining the core language model or fine-tuning the biobank LLM. However, it is important to note that jurisdictional differences in data protection schemes may affect the availability and content of information (e.g., consent forms, protocol details) that can be included in the knowledge base. In such cases, additional adaptation of the RAG model to the local biobank context may be necessary. Nevertheless, RAG remains particularly useful in scenarios where generation needs to be grounded in factual specialized information or where the AI model needs to stay updated with the latest knowledge. In our proposed consent system, the combination of blockchain and a fine-tuned LLM with biobanking RAG would enable demonstrated consent by providing real-time, accurate information in a Q&A style. The biobank AI would be deployed with a user-friendly chat interface capable of answering a wide variety of donor questions involving the use of their samples, research studies, consent preferences, and potential impact on downstream research, while respecting local regulatory requirements.

Importantly, LLM integration could enable the *personalized* provision of information. Just as LLMs can be adapted to generate text in a specific style (Porsdam Mann, Earp, Møller, et al. 2023; Porsdam Mann et al. 2024; Schwitzgebel, Schwitzgebel, and Strasser 2024), so can they be adapted to provide text in a format that is distinctively engaging to, and understandable by, each individual prospective donor (Zohny 2023; Zohny et al. 2024). For instance, this could involve the adaptation of complex consent information into simpler language or the use of analogies, case scenarios, and metaphors to convey the nuances of biobanking research and its potential implications. Such a personalized approach not only demystifies the consent process but also empowers donors to make informed decisions that align with their values and expectations (similar to Earp et al. 2024). By facilitating a deeper understanding of research uses and consent implications, the proposed RAG enhanced LLM would enable donors to make more informed and nuanced consent decisions. This could include specifying conditions under which their samples can be used, understanding the potential for future research developments, or even revisiting and revising consent preferences over time. Accordingly, the proposed demonstrated consent framework, enabled by the combination of generative AI (LLMs) and blockchain technologies, would make possible a more flexible and responsive approach to donor participation in biobanking.

The use of LLMs also introduces efficiencies in the continual consent process. By automating certain aspects of information delivery and consent management, LLMs can reduce the administrative burden on biobanking operations. This not only streamlines the consent process but also allows biobanks to allocate more resources toward their primary mission of supporting biomedical research. Furthermore, the integration of LLMs into demonstrated consent platforms has the potential to foster trust and transparency between donors, biobanks, and researchers. By providing clear, up-to-date, understandable, and detailed information about how samples are being used, LLMs can help bridge the gap between the scientific community and the public.

In sum, by harnessing the capabilities of generative AI LLMs in conjunction with blockchain technology, our proposed demonstrated consent implementation transcends the traditional biobanking consent mechanisms, offering a more transparent and ethically grounded model of engagement that respects individual autonomy while fostering trust and participation in biobanking research.

## DISCUSSION

An ethical consent framework needs to respect individual choice, while also promoting beneficence, respect for persons, and adhering to the demands of justice. To better evaluate these frameworks, Mikkelsen et al. (2019) proposed three criteria: information, value, and duration. To these three criteria we have proposed the addition of a fourth: the balance criterion, which holds that of respect for individual autonomy should be balanced with wider societal interests in the progress of science and medicine, while also honoring the demands of justice.

We introduce AI-enhanced, blockchain-based demonstrated consent as a technical implementation of consent for biobanking. We argue that it best meets the four mentioned criteria because it focuses specifically on the availability of ongoing information, a crucial weakness of previous consent frameworks, without sacrificing the value criterion. In addition, we argue that demonstrated consent most facilitates beneficence and justice, potentially among other moral concerns (e.g., solidarity, the common good), and therefore best fulfills the balance criterion.

Demonstrated consent incorporates the best features of previous proposals. Like meta- and dynamic consent, demonstrated consent involves an initial personalized consent procedure in which the donor expresses their wishes with respect to the uses of their samples for future research. Unlike these prior proposals, demonstrated consent seeks to fulfill the balance criterion by using broad consent as a default or baseline setting. As in broad consent, individuals are given the option to consent to categories or types of research. Those who so desire then have the option of providing further personalized consent modifications such as specifying areas of research in which their samples may not be used or of requesting specific consent for particular uses or at specific intervals. As in dynamic consent, these preferences can later be updated by the individual donor through the blockchain-enabled donor portal or interface. Areas in which no further specifications are made will be treated according to the initial broad consent parameters.

In addition to these features, demonstrated consent leverages the affordances of blockchain and smart contract technology to present near-real time information about the uses to which their samples are being put. This information could include study protocols, grant or IRB applications, associated publications, and ethical provenance documents (Bernier et al. 2023). Donors would not interface directly with these sources of information as their dense and technical nature is likely to hinder understanding by anyone outside the immediate research team. Rather, an LLM system leveraging retrieval-augmented generation would have access to these sources of information. Individual donors would interface with this LLM to gain access to the information they need to make informed consent decisions.

The LLM integration greatly enhances the ongoing availability of information central to our concept of demonstrated consent in two ways. First, it is interactive, allowing individuals to ask follow-up questions and to engage in a process of dialogue likely to enrich their understanding beyond the levels achievable by a static, jargon-laden consent form. Second, it is customizable, enabling individuals to request information in ways that make sense to them. For example, an LLM can be instructed to present information in specific styles or at specific levels of reading comprehension and will do so nearly instantaneously, allowing for many different ways of accessing the information contained in study documentation (Zohny et al., 2024)

These various features and integrations greatly expand the practical ability of consent procedures to fulfill associated major bioethical goals. The ongoing provision of information increases respect for individual autonomy by providing an ongoing, up-to-date, easily accessible and understandable record of the uses to which their samples are put. This in turn is likely to increase trust in biobanking, which will have downstream effects on beneficence and justice. Importantly, study participants may interact solely with the user-friendly LLM interface, which has access to study-specific information, such as consent forms and protocols. The blockchain-based information management system operates entirely on the backend. This approach eliminates the need for participants to directly engage with or understand blockchain technology or NFTs. The ongoing availability of information also removes a major obstacle to the ethical implementation of broad consent. This feature, when combined with a default setting of broad consent, is also likely to increase participation in biobanking compared with other consent architectures.

Importantly, each biological sample associated with an NFT can be de-identified, encrypted, or otherwise pseudonymised in a great variety of ways, given the versatility and composability of blockchain based-smart contracts and biobank tech stack integrations. Structures can be set up such that only the participants themselves can access information about their own samples, maintaining privacy, and allowing for personalized information retrieval.

## LIMITATIONS AND FUTURE DIRECTIONS

The ongoing availability of information concerning the use of one’s samples via demonstrated consent represents, in our view, a significant advancement in how we manage respect for participants in biobanking research. By leveraging new technologies, the framework addresses some of the key weaknesses of previous proposals in allowing participants to make decisions about their continued involvement in research based on up-to-date information.

There are, however, several potential limitations and objections to our proposal which remain to be worked out. First and foremost, our study as reported here does not involve a functioning prototype and we have not provided the kind of technical specificity that would be required to build one. It may therefore well be that unanticipated technical and practical difficulties may arise in the implementation of our proposed system. That being said, the technology behind the proposal is, in our view, at this point sufficiently well understood and developed that it seems likely the proposed platform can be built, and some of us are in the process of attempting to do so.

From an ethical and legal perspective, it could be objected that demonstrated consent threatens donor privacy or data confidentiality. While blockchains are generally regarded as privacy-preserving compared to other transactional systems, there may be concerns about the immutable nature of blockchains and the distribution of information across a decentralized network (theoretically allowing any node to access the information of other nodes). From a regulatory perspective, there are well-known issues concerning immutability of blockchain systems and compliance with the right to be forgotten under the GDPR (Uribe and Waters 2020). These are valid concerns, although some initiatives have already demonstrated how blockchains can address these regulatory technicalities (Mamo et al. 2020; Uribe 2020).

A thorough review of this issue, which is a wider problem affecting blockchain use cases beyond demonstrated consent, is outside the scope of this paper. However, it bears mentioning that there are promising technical solutions on the horizon, including for example the use of zero-knowledge proofs or homomorphic encryption, which are essentially cryptographic methods designed to enhance privacy and security. Zero-knowledge proofs allow parties to prove that they know a value (e.g., the identity of a profile or person associated with a transaction) without revealing the value itself, while homomorphic encryption enables computation on encrypted data without the need to decrypt that data. These techniques may help address compliance complexities by facilitating data privacy and supporting the right to be forgotten, even in immutable blockchain systems (EU Parliamentary Research Services 2019; Corrales, Jurčys, and Kousiouris 2019).

Another possible objection is that there is no guarantee that participants will actively engage with our proposed system, despite theoretical claims that demonstrated consent is expected to improve engagement and transparency. These concerns relate to the fact that people tend to “click-through” digital forms, especially those with complex information, without critically evaluating the information provided (Allen et al. 2024). This is because online environments typically operate using “cognitively frictionless” interfaces designed to reduce cognitive load, and therefore promote attention (Wilbanks 2018). However, demonstrated consent may be less susceptible to “click-through” interactions compared to other online consent models (e.g., dynamic consent). Given its personalized and automatic approach to donor consent, demonstrated consent reduces the amount of unnecessary information communicated during the consent process. Thus, it will be important to perform regular and ongoing reviews of donor satisfaction to ensure that the demonstrated consent process remains effective, user-friendly, and aligned with donors’ expectations and needs.

Environmental concerns have been raised regarding the energy consumption associated with some blockchain technologies (Badea and Mungiu-Pupazan 2021). However, these concerns primarily relate to cryptocurrency applications with vastly higher transaction volumes than would be expected in demonstrated consent. The environmental impact of a demonstrated consent system would depend on its specific implementation, including the chosen consensus mechanism and network architecture. While this is a legitimate concern for proof-of-work architectures, other consensus mechanisms such as proof-of-authority or proof-of-stake are more energy-efficient designs. The use of energy from green data centers among nodes could significantly mitigate ecological effects. Furthermore, any environmental considerations must be weighed against the substantial benefits of demonstrated consent in enhancing participant autonomy, increasing trust in biobanking, and facilitating ethical research practices. As with any new technology, careful attention to sustainability should be part of the design and implementation process.

Additional concerns may relate to the overall approach to demonstrated consent and diminished human oversight compared to conventional approaches to consent in biobanking. While blockchains allow individual donors control over their own data without the need for intermediaries, it may still be necessary to involve human researchers, at least during the initial consent phase, to ensure donors’ voluntary participation from the outset. By updating their consent preferences, donors may also provide subsequent confirmation of their ongoing willingness to use their samples in research. However, future studies could investigate the capacity for LLMs to assess the voluntariness of donors’ consent, thus reducing the need for sustained or primary human involvement in this process.

It is also worth considering the extent to which individual donors should be informed about the technologies involved in demonstrated consent and how their data is being stored and accessed. While donors would not be required to have an in-depth understanding of blockchain or generative AI technology for valid consent, they should still undergo a preliminary consent conversation with a human researcher. In this conversation, researchers would assess both the donor’s willingness to participate in biobanking and their approval to use demonstrated consent processes for the ongoing management of their sample information. This initial consent conversation would allow donors the opportunity to raise any concerns and promote public trust in the consent process.

Finally, further research is needed to explore the scalability of demonstrated consent systems, the interoperability with existing healthcare and research infrastructures, and the long-term impact on participant engagement and trust. Nevertheless, we believe demonstrated consent represents a significant advancement in how we manage and respect donor preferences in biobanking. We argue that demonstrated consent facilitates both donor autonomy and wider societal interests, whilst also optimizing the ethically desirable features of previous consent models.
